# Successful Host Adaptation of IncK2 Plasmids

**DOI:** 10.3389/fmicb.2019.02384

**Published:** 2019-10-15

**Authors:** Marta Rozwandowicz, Michael S. M. Brouwer, Lapo Mughini-Gras, Jaap A. Wagenaar, Bruno Gonzalez-Zorn, Dik J. Mevius, Joost Hordijk

**Affiliations:** ^1^Department of Infectious Diseases and Immunology, Faculty of Veterinary Medicine, Utrecht University, Utrecht, Netherlands; ^2^Wageningen Bioveterinary Research, Lelystad, Netherlands; ^3^National Institute for Public Health and the Environment, Bilthoven, Netherlands; ^4^Faculty of Veterinary Medicine, Institute for Risk Assessment Sciences, Utrecht University, Utrecht, Netherlands; ^5^Antimicrobial Resistance Unit, Department of Animal Health and VISAVET, Complutense University of Madrid, Madrid, Spain

**Keywords:** plasmid, IncK2, conjugation, sigma-32, chicken

## Abstract

The IncK plasmid group can be divided into two separate lineages named IncK1 and IncK2. IncK2 is found predominantly in poultry while IncK1 was reported in various mammals, including animals and humans. The physiological basis of this distinction is not known. In this manuscript we examined fitness cost of IncK1 and IncK2 plasmids at 37 and 42°C, which resembles mammalian and chicken body temperatures, respectively. We analyzed conjugation frequency, plasmid copy number and plasmid fitness cost in direct competition. Additionally, we measured levels of σ-32 in *Escherichia coli* carrying either wild type or conjugation-deficient IncK plasmids. The results show that IncK2 plasmids have a higher conjugation frequency and lower copy number at 42°C compared to IncK1. While the overall fitness cost to the host bacterium of IncK2 plasmids was higher than that of IncK1, it was not affected by the temperature while the fitness cost of IncK1 was shown to increase at 42°C compared to 37°C. These differences correlate with an increased expression of σ-32, a regulator of heat-shock protein expression, in *E. coli* with IncK2 compared to cells containing IncK1. This effect was not seen in cells containing conjugation deficient plasmids. Therefore, it is hypothesized that the assembly of the functional T4S may lead to these increased levels of σ–32. Increased activation of CpxR at 42°C may explain why IncK2 plasmids, and not IncK1, are predominantly found in chicken isolates.

## Introduction

Antimicrobial resistance is a global health threat and was responsible for an estimated 33110 infection-related deaths in European Union in 2015 (Cassini et al., 2019). As antimicrobial resistance (AMR) is often encoded on plasmids, it is crucial to understand the dynamics of plasmid spread. One of the determinants influencing plasmid spread is plasmid fitness cost, which is defined as a burden on the bacterial host, manifesting in reduced growth rate and weakened competitiveness of plasmid-bearing strains under conditions that do not select for plasmid-encoded genes (Vogwill and MacLean, 2015). Plasmid fitness cost can derive from many processes. Entrance into the cell triggers an SOS response which may delay cell division (Ingmer et al., 2001). Moreover, replication causes depletion of essential cellular components like RNA polymerase, tRNA and amino acids (Wegrzyn and Wegrzyn, 2002). A recent review of San Millan and Maclean describes these mechanisms in greater detail (San Millan and MacLean, 2017).

Carrying a plasmid may not only be a burden, but also provide a certain evolutionary advantage (Enne et al., 2004; Yates et al., 2006; Michon et al., 2011). It was shown that plasmids entering a new host, where they initially pose a fitness cost, after short-term evolution, become advantageous (Dionisio et al., 2005). This phenomenon counteracts efforts to lower AMR levels as it seems that plasmids can persist in their host even without selective pressure.

The fact that plasmids can show a high stability in bacterial populations in the absence of apparent selective pressure is counter intuitive to the fitness burden that they impose on the bacterial host cell, this is also referred to as the plasmid paradox (San Millan and MacLean, 2017). With the advancement of studies on plasmid fitness cost it became clear that plasmids evolved strategies to neutralize their fitness cost. Based on many plasmid fitness cost studies described in literature, Harrison and Brockhurst (2012) pointed out that simultaneous evolution of plasmid and its host (coevolution) is the most important factor in reducing fitness cost. The phenomenon of coevolution partly resolves the plasmid paradox. Another way to neutralize plasmid fitness cost is compensatory evolution. Plasmids posing a high fitness cost, can minimize it through evolutionary changes induced by the bacterial host (Harrison et al., 2016). One example is mutations in the CheY protein of *Pseudomonas moraviensis*, which were reported to decrease plasmid fitness cost (Loftie-Eaton et al., 2016). Another example reported to explain a decrease in plasmid fitness cost is an IS-mediated deletion of a 25 kb plasmid fragment containing three resistance determinants, a restriction anti-restriction system, and the main conjugation machinery (Porse et al., 2016). Moreover, it was shown for ColE plasmids that compensatory evolution enables coexistence of multiple copies of a plasmid (Santos-Lopez et al., 2017b). Another strategy is gene silencing, which prevents expression of xenogeneic DNA, which in turn can be harmful to the bacterial cell or cause severe fitness consequences (Navarre, 2016). Expression of these genes can be switched on in certain environmental conditions. Gene silencing can be mediated by H-NS or plasmid partitioning proteins. H-NS proteins act as a master regulator, which can affect transcription of up to 60 genes in *Escherichia coli* (Hommais et al., 2001). Silencing of only resistance genes was also reported (Enne et al., 2006). Silencing of genes due to the partition proteins was reported for F and P1 plasmids (Lynch and Wang, 1995; Rodionov et al., 1999). Compensatory mutations to reduce fitness cost can result in converging evolution of plasmids that were once closely related and can result in adaptation to specific niches.

It was shown that IncK plasmids can be divided into two separate lineages named IncK1 and IncK2 (Rozwandowicz et al., 2017; Seiffert et al., 2017). IncK2 is found predominantly in poultry sources while IncK1 was reported in various animal and human sources. A possible explanation could be adaptation of IncK2 plasmids to poultry specific characteristics like e.g., a slightly higher body temperature compared to other animals and humans.

Environmental temperature is known to have major effects on bacterial evolution, which is also influenced by the body temperature of a colonized host (Dawoud et al., 2017). Most research focuses on temperatures lower than 37°C, mimicking environmental or food storage conditions (Paytubi et al., 2014; Mo et al., 2017). There is only limited data available on plasmid fitness cost in elevated temperatures. The body temperature of chickens is 42°C vs. 37–39°C of mammals, depending on the animal species involved. The higher chicken body temperature was demonstrated to induce a heat-shock response for optimal fitness of *Salmonella* residing in chicken ceca (Troxell, 2016). A heat-shock response can further influence plasmid fitness cost by increasing conjugation frequency or biofilm formation (Mellata et al., 2012; Zeng et al., 2015; Kirk and Fagan, 2016). Zahrl et al. (2006, 2007) showed that assembly of T4S triggers activation of the extracytoplasmic stress, which is sensed by the two-component system CpxRA. That leads to increased levels of σ-32, which in turn is responsible for the heat-shock response. These findings suggest that elevated temperatures may play an important role in plasmid adaptation to the animal host.

In recent years several methods to measure plasmid fitness cost were developed. The most widely used *in vitro* experiments focus on bacterial growth and direct competition between plasmid-bearing and plasmid-free strains (Petersen et al., 2009; Gottig et al., 2016; Gumpert et al., 2017). Furthermore, a mouse model was used to assess plasmid fitness cost *in vivo* (Gumpert et al., 2017). Fitness cost can also be assessed indirectly by measuring the conjugation rate or the rate of biofilm formation (Mellata et al., 2012).

The goal of this research was to examine the fitness cost of IncK1 and IncK2 plasmids on its bacterial host. To achieve that, growth rates, conjugation frequency, direct competition and plasmid copy numbers were determined at 37 and 42°C. Additionally, we determined levels of σ-32 in *E. coli* with and without the presence of IncK plasmids.

## Materials and Methods

### Plasmids and Bacterial Strains

In this study, we used IncK1 plasmids p754 and p527, isolated from *E. coli* obtained from a dog and cattle, respectively. The IncK2 plasmids pT.1.09 and pT.10.2, isolated from *E. coli* obtained from poultry. The IncK1 plasmids used in this study carry *bla*_CTX–M–14_, while the IncK2 plasmids carry *bla*_CMY–2_. The *E. coli* MG1655 strain, used as recipient for conjugation experiments, encodes resistance to chloramphenicol. All experiments were performed at 37 and 42°C. These temperatures were chosen to resemble the body temperatures of mammals and chickens, respectively.

### Conjugation Rate

Conjugation was performed as previously described (Rozwandowicz et al., 2017). Briefly, liquid cultures of donor and recipient cells at OD_600_ 0.5 were mixed in 1:1 ratio and incubated for 18 h at 37 or 42°C. Donor and transconjugant cells were recovered on LB plates or LB plates supplemented with 2 mg/L cefotaxime and 25 mg/L chloramphenicol, respectively. All experiments were performed in triplicate. Conjugation frequency was calculated as the number of transconjugants per donor cell. Obtained data was analyzed using the Mann-Whitney test with *p* > 0.05 considered statistically significant.

### *traY* Gene Mutagenesis

To obtain a non-conjugative IncK plasmid for competition experiments, a kanamycin resistance gene was knocked-in to the *traY* gene using the RedET system (Gene Bridges). Primers used for the mutagenesis are listed in Supplementary Table S1. Mutagenesis was performed according to the kit protocol. Insertion of the resistance cassette in the *traY* gene was confirmed by PCR using primers “K1 traY fw” and “K1 traY rv” for IncK1 plasmids and “K2 traY fw” and “K2 traY rv” for IncK2 (Supplementary Table S1).

### Stability and Competition

The stability of the mutant plasmids, as well as the gene insertions, were measured over the course of five consecutive days without selection. 50 μl of bacterial suspension with a density 0.5 McFarland was incubated in 5 mL LB broth and grown at 37 or 42°C. Cultures were renewed daily in fresh LB in 1:1000 ratio after overnight incubation for 5 days. Samples of the overnight cultures were plated on LB plates and after overnight incubation 100 separate colonies were replicated on LB plates with 2 mg/L cefotaxime or 15 mg/L kanamycin.

Competition experiments between *E. coli* DH10B and *E. coli* DH10B IncK1-traY::kan, or *E. coli* DH10B and *E. coli* DH10B IncK2-traY::kan, were performed in triplicate. The procedure to test the fitness cost was the same as the stability tests, but using multiple strains in competition. Fitness cost was calculated as previously described (Santos-Lopez et al., 2017a). Briefly, the CI (competition index) was calculated as the ratio of the mean cfu for three independent competition experiments between the resistant and susceptible strains at a given time point (*t*_1_) divided by the same ratio at *t*_0_. The selection coefficient, *s*, was calculated as the slope of the linear regression model: *s* = ln(CI)/ln(*d*), where *d* is the dilution factor. The selection coefficient estimates the difference between the relative fitnesses of the two competitors over the entire competition experiment. The relative fitness (*w*) was calculated with the formula *w* = 1 + *s*. Obtained data were analyzed using the Mann-Whitney test.

### Plasmid Copy Number

To determine the plasmid copy number, three independent DNA extractions using QIAamp DNA Midi Kit (Qiagen) were performed for each strain, and qPCRs (BioRad), targeting the IncK replicon and uidA gene, were carried out in triplicate for each extraction. Plasmid copy number per chromosome was calculated using the formula described by San Millan et al. (2015) c_n_ = [(1 + E_c_)^Ctc^/(1 + E_p_)^Ctp^] x (S_c_/S_p_), where c_n_ is the plasmid copy number per chromosome, S_c_ and S_p_ are the sizes of the chromosomal and plasmid amplicons (in bp), respectively, E_c_ and E_p_ are the efficiencies of the chromosomal and plasmid qPCRs (relative to 1), respectively, and Ctc and Ctp are the threshold cycles of the chromosomal and plasmid reactions, respectively. Plasmid copy number was determined at 37 and 42°C using K/B fv and K rv primers for IncK2, K/B fv new and K rv new primers for IncK1 and uidA fw and uidA rv for the chromosomal target (Supplementary Table S1). Obtained data were analyzed using the Mann-Whitney test.

### Sigma-32 Levels Analysis

σ-32 levels were analyzed in whole-cell lysates corresponding to 0.3 OD_600_. 4 mL of the culture was centrifuged at 3500 × *g* for 1 min. The pellets were resuspended in 100 μL of the protease inhibitor mixture (cOmplete^TM^, Mini Protease Inhibitor Cocktail, Roche). 15 uL of the samples were loaded on 12% polyacrylamide gels. Immunological detection of σ-32 was performed using a monoclonal anti-σ-32 antibody (Neoclone), diluted 1:2000 in PBS buffer with 0.5% gelatin, and 0.1% Triton x-100 (Sigma Aldrich). As secondary antibodies, HRP conjugated goat anti-mouse IgG antibodies (Sigma Aldrich) diluted 1:8000 in PBS buffer with 0.5% gelatin and 0.1% Triton x-100 were used. The chemiluminescent detection was performed using the Clarity ECL system (Bio Rad). As a positive control for the specificity of the antibodies, a strain with mutated σ-32 was used (Nagai et al., 1991) *rpoH*-*lacZ* gene fusion results in higher molecular weight of the protein, which enables correct identification with western blot. σ-32 protein levels were standardized to the *E. coli* DH10 sample at 37 or 42°C. The relative quantity of σ-32 is an average of three independent experiments. Obtained data were analyzed using the Mann-Whitney test.

## Results

### Conjugation Rate

**FIGURE 1:**
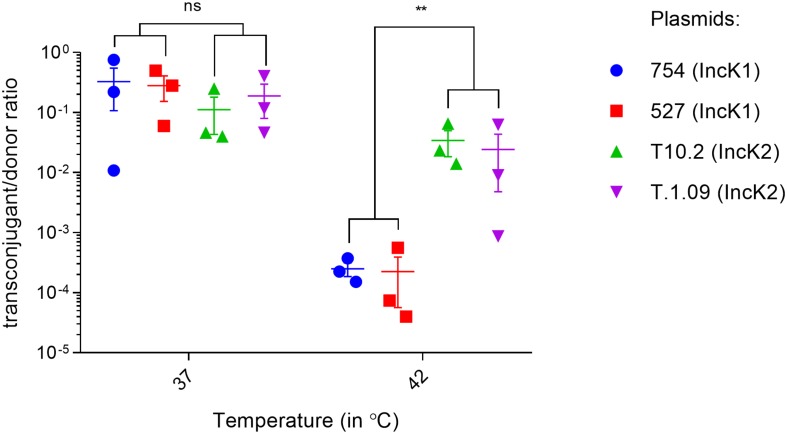
Triplicate measures of conjugation rates of IncK plasmids at 37 and 42°C. Bars depict the mean and standard deviation. ^∗∗^ depict statistical significance (*p* = 0.0039).

Conjugation rate experiments in liquid broth matings were performed at 37 and 42°C to assess differences in the spread potential of IncK1 and IncK2 plasmids. At 37°C there was no difference in conjugation rate between IncK1 and IncK2 plasmids (Figure 1). At 42°C, the rate of both plasmid types significantly decreased compared to 37°C. However, the rate of IncK1 was decreased much further than that of the IncK2 plasmids, compared to 37°C. At 42°C there is a significant difference in conjugation rate between IncK1 and IncK2 plasmids (*p* = 0.0039) (Figure 1).

### Plasmid Copy Number

**FIGURE 2:**
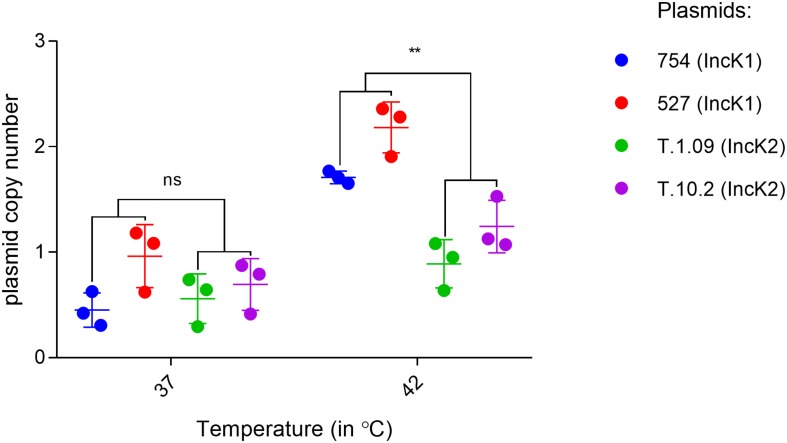
IncK1 and IncK2 plasmid copy numbers at 37 and 42°C. All the measurements were performed in triplicate. Bars depict the mean and standard deviation. ^∗∗^ depict statistical significance (*p* = 0.0039).

The plasmid copy number of IncK1 and IncK2 plasmids in *E. coli* DH10 were assessed at 37 and 42°C. For both of the IncK1 and IncK2 plasmids that were tested here, the plasmid copy number at 37°C was consistently just under 1 copy per chromosome, reflecting a bias in the DNA isolation method that was used (Becker et al., 2016). Assuming that this bias is equal for all isolates, all plasmids, besides T.109, showed a significant difference in copy number at 37 and 42°C. The difference in copy number between IncK1 and IncK2 plasmids at 42°C was statistically significant (*p* = 0.0039), while there was no difference at 37°C (Figure 2). Both IncK1 and IncK2 plasmids showed a statistically significant increase in copy number at 42°C compared to 37°C (*p* = 0.0039 and *p* = 0.0250, respectively).

### Fitness Cost

To make IncK plasmids non-transmissible, knock-in mutants were created by inserting a kanamycin resistance cassette into the *traY* gene (Cottell et al., 2011). Conjugation experiments were performed to confirm the inability of conjugative transfer of IncK plasmids (data not shown). Stability of the IncK plasmid with the insertion in the *traY* gene was confirmed during the competition experiment. The *E. coli* DH10B IncK-traY::kan stably maintained these plasmids throughout the duration of the experiment.

**FIGURE 3:**
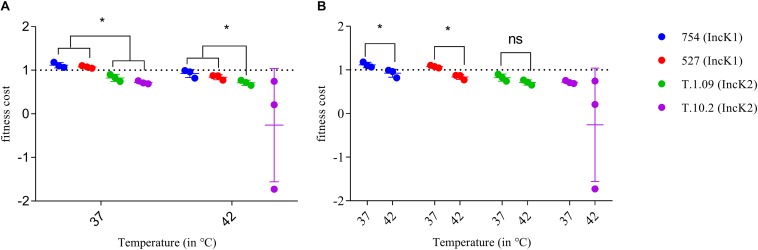
Fitness cost of IncK1 and IncK2 plasmids measured at 37 and 42°C. All the measurements were performed in triplicate. Bars depict the mean and standard deviation. **(A)** Comparison of plasmid fitness cost by temperature. ^∗^ depict statistical significance (*p* = 0.0201). **(B)** Comparison of plasmid fitness cost by plasmid type. ^∗^ depict statistical significance (*p* = 0.0495).

At both 37 and 42°C IncK1 plasmids had a lower fitness cost compared to IncK2 plasmids (T.1.09) (*p* = 0.0201) (Figure 3A). Due to a large standard deviation at 42°C, plasmid T.10.2 was excluded from the statistical analysis. Differences between 37 and 42°C were significant for IncK1 plasmids (*p* = 0.0495), but not for IncK2 (Figure 3B).

### Sigma-32 Levels

**FIGURE 4:**
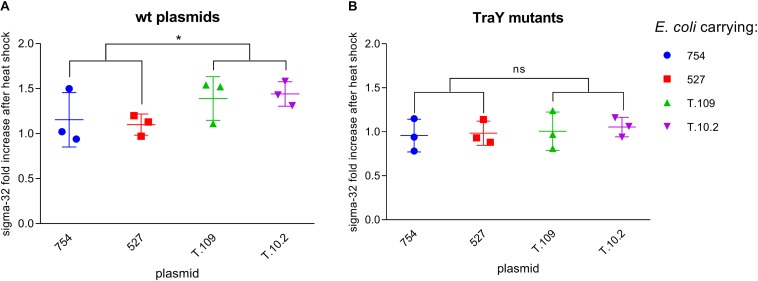
**(A)** σ-32 fold increase between 37 and 42°C in *Escherichia coli* carrying wt plasmids. ^∗^ depict statistical significance (*p* = 0.037). **(B)** σ-32 fold increase between 37 and 42°C in *E. coli* carrying plasmids with mutated TraY; Bars depict the mean and standard deviation.

The levels of σ-32 in lysates of *E. coli* cell suspensions at OD 0.3, with or without IncK1 or IncK2 plasmids were measured. In our experiment, a statistically significant difference (*p* = 0.037) in σ-32 upshift between 37 and 42°C between IncK1 and IncK2 plasmid carrying *E. coli* was observed (Figure 4). The difference in σ-32 upshift between 37 and 42°C was not observed when testing non-transmissible IncK plasmids (IncK-traY::kan).

## Discussion

While most attention in previous studies has focussed on plasmid behavior at temperatures below mammalian body temperature, it is known that elevated temperatures can lead to a higher conjugation rate, biofilm formation, and a higher plasmid copy number per cell (Hall and Vockler, 1987; Bertrand-Burggraf et al., 1989; Kapralek et al., 1998; Mellata et al., 2012; Zeng et al., 2015; Kirk and Fagan, 2016).

In the present study we have examined various attributes of IncK1 and IncK2 at both 37 and 42°C to resemble the mammalian and chicken body temperature, as these are the respective niches of IncK1 and IncK2 plasmids (Rozwandowicz et al., 2017; Seiffert et al., 2017). These attributes included several well studied properties including the conjugation frequency, plasmid copy number and plasmid fitness cost of plasmids from the IncK group, as well as the levels of expression of σ-32 to assess the extra-cytoplasmic stress level induced by the plasmid conjugation. All of these experiments were performed with the IncK1 plasmids p754 and p527 isolated from dog and cattle, respectively, and the IncK2 plasmids pT.1.09 and pT.10.2 isolated from poultry.

Conjugative plasmids pose a difficulty during direct competition as it is impossible to differentiate between cells that originally had a plasmid and the ones that received it via conjugation during the experiment. One of the solutions can be to introduce mutations in the conjugation pathway of a plasmid. It was shown that mutation of the *traY* gene of IncK plasmids effectively prevents conjugation (Cottell et al., 2014).

Measurement of the plasmid copy number at 37°C resulted in a consistent copy number just below 1 copy per chromosome for all four plasmids that were tested here. As all cells were grown on selective media before the experiment, an actual copy number below 1 copy per chromosome is likely caused by a bias in the DNA isolation method (Becker et al., 2016). Nonetheless, this bias is presumed equal for all plasmids that were tested and differences in the relative abundance of plasmids can still be compared between the samples. At 37°C there are no differences in plasmid copy number between IncK1 and IncK2 plasmids but at 42°C IncK1 plasmids have statistically significant higher copy numbers compared to IncK2 plasmids.

The method that was used to measure the burden on the fitness cost of the host bacterium was previously described by Santos-Lopez and uses direct competition between bacteria with and without a plasmid (Santos-Lopez et al., 2017a). At 37°C, both of the IncK1 plasmids have a small fitness advantage for the *E. coli* while IncK2 plasmids present a burden. Although these differences are minor, there is a significant effect which may lead to selection of IncK1 over IncK2 *in vivo* at this temperature. At 42°C, the IncK1 plasmid also has a lower fitness cost compared to the IncK2 plasmid. Looking at each plasmid separately, IncK1 plasmids have a significantly higher fitness cost at 42°C compared to 37°C, while for IncK2 plasmids this difference is not significant.

In this manuscript we measured fitness cost of IncK plasmids in an indirect as well as a direct manner. Conjugation rate and copy number are examples of the plasmid’s characteristics that indirectly influence the fitness cost the plasmid causes to the host. By performing competition experiments we demonstrate in a direct way the burden of carrying an IncK plasmid for the *E. coli* host. We believe that measuring multiple fitness cost related parameters will allow for a better understanding of plasmid fitness cost.

Additionally, to determine a potential stress response we measured levels of σ-32 in *E. coli* carrying either IncK1 or IncK2 plasmids. The IncK2 plasmids causes a higher upshift of σ-32 levels, compared to the IncK1 plasmids. This effect is no longer observed when the *traY* gene is disabled by creating a knock-in mutant, which inactivates the assembly of the conjugation machinery. These results are in line with the model proposed by Zahrl et al. (2006) which links conjugation to a heat shock response, which was manifested by elevated levels of σ-32. In their model, the assembly of a functional TS4-system generated a stress signal which is sensed by CpxRA envelope stress signaling system, which subsequently leads to a transcriptional induction of both extra-cytoplasmic stress genes as well as the *rpoH* gene (encoding σ-32).

The model proposed by Zahrl et al. (2006) demonstrates that a lower conjugation rate of both IncK1 and IncK2 plasmids at 42°C compared to 37°C was associated with cytoplasmic stress. This suggests that cytoplasmic stress caused by the high level of σ-32 at 42°C decreased the conjugation rate. Upregulation of σ-32 in IncK1-carrying cells leads to the cytoplasmic stress, which was proven to decrease plasmid conjugation. These findings explain lower conjugation level of IncK1 plasmid compared to IncK2 at 42°C.

The role of the two-component system CpxRA in bacterial virulence was previously discussed in literature, with conflicting results. Some groups reported the role of CpxRA in pathogenesis through modulation of expression of virulence factors and regulators (Nakayama and Watanabe, 1998; Mitobe et al., 2005; Nevesinjac and Raivio, 2005; Humphries et al., 2010; Thomassin et al., 2015). Others showed that activation of CpxRA inhibits the virulence (Carlsson et al., 2007; Macritchie et al., 2008). Vogt et al. (2010) reported that depending on the level of induction of Cpx response, it can promote or inhibit the assembly of *E. coli* bundle-forming pilus. More recent research has shown that CpxR plays a crucial role in regulation of genes important for colonization of *Salmonella* and avian pathogenic *E. coli* (APEC) (Matter et al., 2018; Subramaniam et al., 2019).

**FIGURE 5:**
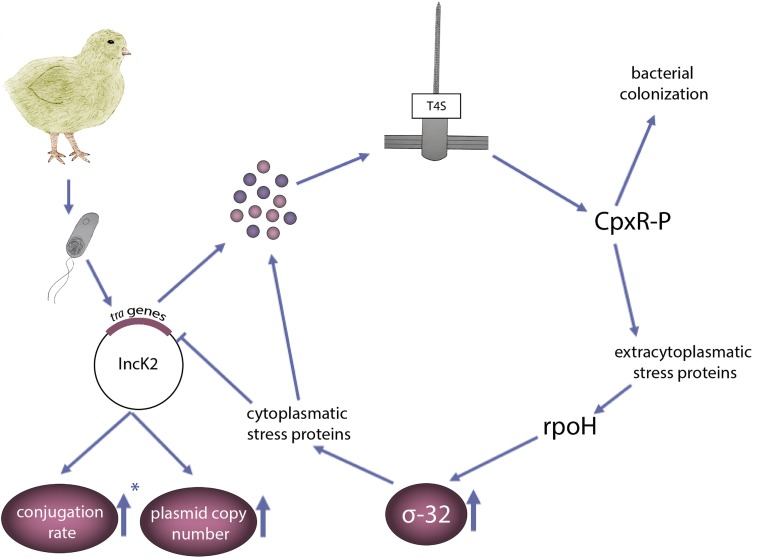
Schematic presentation of the environmental context to the plasmid induced bacterial stress model adapted from Zahrl et al. (2006), which explains the high prevalence of IncK2 (and not IncK1) in poultry isolates. *E. coli* carrying an IncK2 plasmid that enters the chicken gut expresses the tra proteins. Assembly of a T4S induces extracytoplasmic stress sensed by CpxR. CpxR, which was associated with increased bacterial colonization, induces the σ-32 response encoded by *rpoH* gene, resulting in a further upregulation of cytoplasmic stress proteins. ^∗^Arrow pointing up means less decrease in conjugation rate of IncK2 plasmid compared to IncK1 at 42°C.

The results presented in this study add environmental context to the plasmid induced bacterial stress model described by Zahrl et al. (2006) (Figure 5). Bacteria carrying an IncK2 plasmid that enters the chicken body, have a higher conjugation rate compared to an IncK1 plasmid, which results in higher expression and assembly of T4S. Using conjugation deficient mutants confirmed the role of increased conjugation rate of IncK2 plasmid in the presented pathway. Assembly of T4S leads to extracytoplasmic stress, which is sensed by CpxRA. Activation of the Cpx response leads to increased levels of σ-32. Increased levels of CpxRA were reported to have a role in colonization of *Salmonella* and *E. coli* APEC (Matter et al., 2018; Subramaniam et al., 2019). All of the above data leads to the possible explanation why IncK2, and not IncK1 plasmids, are predominantly found in the chicken isolates by promoting a fitness advantage to the host bacterium at relatively high temperatures.

Obtained results show how chicken’s body temperature influences plasmid fitness cost in *E. coli* host. Increased temperature leads to higher conjugation rate of IncK2 plasmids, which by inducing stress response, activates CpxRA which was proven to be involved in bacterial colonization. Fitness cost of IncK2 plasmid, in contrast to IncK1 plasmid, is not changed with higher temperature. Moreover, IncK1 plasmid copy number is abnormally high at the chicken body temperature, which may lead to the instability. These data shed a light on IncK2, and not IncK1, plasmid’s success in invading chicken and gives a possible target to eliminate these plasmids from chicken isolates.

## Data Availability Statement

All datasets generated for this study are included in the manuscript/Supplementary Files.

## Ethics Statement

Faecal samples were taken either after excretion by the animal or by rectal swabs. All sampling falls within the guidelines of the Dutch Animals Act (https://zoek.officielebekendmakingen.nl/stb-2011-345.html) and the Animal Welfare Body Utrecht (http://www.ivd-utrecht.nl/en/), meaning no additional ethics approval was required.

## Author Contributions

MB, JW, BG-Z, DM, and JH designed the study, while MR and MB designed the laboratory experiments that were performed by MR. LM-G analyzed the data statistically. MR wrote the original draft. MB, LM-G, JW, BG-Z, DM, and JH critically reviewed the manuscript.

## Conflict of Interest

The authors declare that the research was conducted in the absence of any commercial or financial relationships that could be construed as a potential conflict of interest.
