# Supplementary material for: Successful Host Adaptation of IncK2 Plasmids
Source: Front Microbiol. 2019 Oct 15;10:2384. doi: 10.3389/fmicb.2019.02384 (PMC6803427; doi:10.3389/fmicb.2019.02384)
Supplement: Supplementary file 1 [file Table_1.DOCX]

Table S1 Primers used in the study

| Primer name | Primer sequence | Reference |
| --- | --- | --- |
| TraY-homology1-Fw | tacaggcgcgggcataaatcatcaaaaatcccgccatgctgaacacgcccgtcacagagtttattaactaactagTGGACAGCAAGCGAACCGGAATTGC | this study |
| TraY-homology1-Rev | agtcagcgtactgattggatgtgctgctggccccctgtgggccgccacccatctgggaacaagtcaTCAGAAGAACTCGTCAAGAAGGCG | this study |
| TraY-homology2-Fw | gccattctctgtccgtgaggattcgctcatgggcggcgttgatgaacgtgcggtacggttaaagtaactaactagTGGACAGCAAGCGAACCGGAATTGC | this study |
| TraY-homology2-Rev | tggcgcaactggtcatgctgtggggcgcatccatcatgggcatcggttccgccaatatcatggttTCAGAAGAACTCGTCAAGAAGGCG | this study |
| TraY-TS1-Fw | ACGCCCGTCACAGAGTTTAT | this study |
| TraY-TS1-Rev | ACCCATCTGGGAACAAGTCA | this study |
| TraY-TS2-Fw | AACGTGCGGTACGGTTAAAG | this study |
| TraY-TS2-Rev | GTTCCGCCAATATCATGGTT | this study |
| K1 traY rv | GCAATTATCGCCACGATGGG | this study |
| K2 traY fw | ACAGACGCGGGCATAAATCA | this study |
| K2 traY rv | GACCGGCATTGGTAACTGGA | this study |
| uidA fw | GTCAATAATCAGGAAGTG | this study |
| uidA rv | AAAGAAATCATGGAAGTAA | this study |
| K/B fv | GCGGTCCGGAAAGCCAGAAAAC | Carattoli *et al. (1)* |
| K rv | TCTTTCACGAGCCCGCCAAA | Carattoli *et al. (1)* |
| K/B fv new | AGGATCCGGGAAGTCAGAAAAC | Rozwandowicz *et al.* (2) |
| K rv new | TCTTTCACGATCCCGCCAAA | Rozwandowicz *et al.* (2) |

References

1. Carattoli A, Bertini A, Villa L, Falbo V, Hopkins KL, Threlfall EJ. Identification of plasmids by PCR-based replicon typing. *J Microbiol Methods* (2005) **63**:219-28 doi: S0167-7012(05)00113-2 [pii].

2. Rozwandowicz M, Brouwer MS, Zomer AL, Bossers A, Harders F, Mevius DJ, et al. Plasmids of Distinct IncK Lineages Show Compatible Phenotypes. *Antimicrob Agents Chemother* (2017) **61**:10.1128/AAC.01954,16. Print 2017 Mar doi: e01954-16 [pii].
